# Selenoproteins Protect Against Avian Liver Necrosis by Metabolizing Peroxides and Regulating Receptor Interacting Serine Threonine Kinase 1/Receptor Interacting Serine Threonine Kinase 3/Mixed Lineage Kinase Domain-Like and Mitogen-Activated Protein Kinase Signaling

**DOI:** 10.3389/fphys.2021.696256

**Published:** 2021-08-12

**Authors:** Tong Li, Jing Zhang, Peng-Jie Wang, Zi-Wei Zhang, Jia-Qiang Huang

**Affiliations:** ^1^Beijing Advanced Innovation Center for Food Nutrition and Human Health, Department of Nutrition and Health, China Agricultural University, Beijing, China; ^2^Administrative Engineering College, Xu Zhou University of Technology, Xuzhou, China; ^3^Key Laboratory of Precision Nutrition and Food Quality, Ministry of Education, Department of Nutrition and Health, China Agricultural University, Beijing, China; ^4^College of Veterinary Medicine, Northeast Agricultural University, Harbin, China

**Keywords:** selenium, chick, selenoprotein, signaling pathway, liver necrosis

## Abstract

Liver necroptosis of chicks is induced by selenium (Se)/vitamin E (VE) deficiencies and may be associated with oxidative cell damage. To reveal the underlying mechanisms of liver necrosis, a pool of the corn–soy basal diet (10 μg Se/kg; no VE added), a basal diet plus all-rac*-*α-tocopheryl acetate (50 mg/kg), Se (sodium selenite at 0.3 mg/kg), or both of these nutrients were provided to day-old broiler chicks (*n* = 40/group) for 6 weeks. High incidences of liver necrosis (30%) of chicks were induced by –SE–VE, starting at day 16. The Se concentration in liver and glutathione peroxidase (GPX) activity were decreased (*P* < 0.05) by dietary Se deficiency. Meanwhile, Se deficiency elevated malondialdehyde content and decreased superoxide dismutase (SOD) activity in the liver at weeks 2 and 4. Chicks fed with the two Se-deficient diets showed lower (*P* < 0.05) hepatic mRNA expression of *Gpx1, Gpx3, Gpx4, Selenof, Selenoh, Selenok, Selenom, Selenon, Selenoo, Selenop, Selenot, Selenou, Selenow*, and *Dio1* than those fed with the two Se-supplemented diets. Dietary Se deficiency had elevated (*P* < 0.05) the expression of SELENOP, but decreased the downregulation (*P* < 0.05) of GPX1, GPX4, SELENON, and SELENOW in the liver of chicks at two time points. Meanwhile, dietary Se deficiency upregulated (*P* < 0.05) the abundance of hepatic proteins of p38 mitogen-activated protein kinase, phospho-p38 mitogen-activated protein kinase, c-Jun N-terminal kinase, phospho-c-Jun N-terminal kinase, extracellular signal-regulated kinase, phospho-mitogen-activated protein kinase, receptor-interacting serine-threonine kinase 1 (RIPK1), receptor-interacting serine-threonine kinase 3 (RIPK3), and mixed lineage kinase domain-like (MLKL) at two time points. In conclusion, our data confirmed the differential regulation of dietary Se deficiency on several key selenoproteins, the RIPK1/RIPK3/MLKL, and mitogen-activated protein kinase signaling pathway in chicks and identified new molecular clues for understanding the etiology of nutritional liver necrosis.

## Introduction

The recognized sign of selenium (Se) deficiency, liver necrosis, was mostly discovered in rats (Burk et al., 2010; Schwarz and Foltz, 2010; Wang H. et al., 2020) and swines (Tang et al., 2020; Zhang Y. et al., 2020). However, in our previous report (Huang et al., 2011, 2015), we also found liver necrosis induced by Se and vitamin E (VE) deficiency in chicks. We have investigated the expression profiles of 14 selenoprotein genes (i.e., *Gpx1, Gpx4, Selenof*, *Selenoi, Selenok, Selenon, Selenoo, Selenop, Selenos, Selenot, Selenow, Msrb1, Txnrd1*, and *Txnrd2*) in the liver and muscles of chicks fed with Se-deficient (0.01 mg/kg) and adequate (0.3 mg/kg) diets at week 6 (Huang et al., 2011, 2015). Dietary Se deficiency decreased (*P* < 0.05) mRNA levels of seven common selenoprotein genes (i.e., *Gpx1, Gpx4, Selenok, Selenon, Selenoo, Selenop*, and *Selenow*) in liver and muscle. Nevertheless, these are the genetic changes far from after the onset of liver necrosis (Ren et al., 2018). Recently, 24–25 selenoprotein genes were postulated in avians (Mariotti et al., 2012; Li et al., 2018), so this study followed to investigate the changes of 25 selenoproteins in the liver of chicks responded to dietary Se and VE deficiencies. The fact reminds us that the expression between proteins and genes is not identical; sometimes, the expression of the proteins is closer to the physiological condition (Alan, 2015; Li and Sunde, 2016; Katarzyna et al., 2020). Therefore, we assayed seven key selenoproteins (i.e., GPX1, GPX3, GPX4, SELENON, SELENOP, SELENOW, and MSRB1) in liver. The activities and mRNA levels of two abundant selenoprotein (GPX1 and GPX4) genes are highly regulated by Se status in chicks (Huang et al., 2011; Li et al., 2018; Ren et al., 2018). Meanwhile, other five selenoprotein genes code for glutathione peroxidase 3 (GPX3) (Zhu et al., 2018), selenoprotein P (SELENOP) (Short et al., 2020; Gül-Klein et al., 2021), selenoprotein W (SELENOW) (Shin et al., 2019), selenoprotein N (SELENON) (Silwal et al., 2020), and methionine sulfoxide reductase B1 (MsrB1) were also regulated by Se status in chicks.

Both apoptosis and necrosis frequently occur together after death signals and toxic stresses (Xiao et al., 2015; Seremelis et al., 2019). The term, necrapoptosis, describes such a process of death that begins with a general stress or death signal (Amr et al., 2004). Necroptosis, a newly discovered pathway, regulated necrosis and mediated by the proteins of receptor-interacting protein kinase-1 (RIPK1), RIPK3, and mixed lineage kinase domain-like (MLKL) (Wang Y. et al., 2020). Also, the cleavage of RIPK1 and RIPK3 has been identified as the most important steps in the prevention of necroptosis (Yan et al., 2018; Iorga et al., 2021; Liu et al., 2021). Some studies have suggested that integrin activation of phosphatidylinositol-3-kinase (PI3-kinase), c-Jun NH2-terminal kinase (JNK), and extracellular signal-regulated kinase (ERK) signaling pathways require focal adhesion kinase (FAK) (Huang et al., 2015). The p38 mitogen-activated protein kinase (MAPK) and JNK pathways play an important role in ROS-induced apoptosis. It has been shown that prevention of p38 MAPK phosphorylation at Thr-180/Tyr-182 under hypoxia-induced oxidative stress in mouse embryonic fibroblasts due to the overexpression of GPX1 (Huang et al., 2018). The first report on liver necrosis caused by Se deficiency can date back to 1957. It was the first report showing that Se is essential for mammals, specifically rats (Burk et al., 2010; Schwarz and Foltz, 2010). Although liver injury and Se deficiency have been studied extensively, less is known about how these protein kinases are associated with avian liver necrosis. Therefore, we conducted this experiment to (1) compare the effects of Se and VE on mRNA expression profiles of 25 selenoproteins in liver of chicks at week 2 prior to the onset of liver necrosis, (2) explore how tissue selenoprotein levels of GPX1, GPX3, GPX4, SELENOP, SELENOW, SELENON, and MSRB1 in liver of chicks were related to liver necrosis, and (3) determine the responses of RIPK1/RIPK3/MLKL and MAPK signaling pathway proteins in chicks associated with liver necrosis.

## Materials and Methods

### Animal, Diet, and Experimental Design

Our animal protocol was approved by China Agricultural University. A total of 160 day-old male broiler chicks (Dafa Zhengda Poultry Co., Ltd., Beijing, China) were selected and allotted into 4 dietary treatment groups (*n* = 40). Each group of 40 chicks was kept in stainless-steel cages (length × width × height, 2 m × 2 m × 50 cm). The basal diet (Table 1) was composed of corn and soybean produced in the Se-deficient area of Sichuan, China and was not supplemented with Se or VE (–SE–VE). The other three experimental diets were supplemented with rac-α-tocopheryl acetate (PureOne Biotechnology, Shanghai) at 50 mg/kg (–SE+VE), Se (as sodium selenite, Sigma-Aldrich, St Louis, MO, USA) at 0.3 mg/kg (+SE–VE), or both of these nutrients (+SE+VE). The analyzed Se concentrations were 11, 15, 351, and 342 μg/kg of feed (as fed) for these four diets, respectively. Chicks were housed in battery brooder cages with raised wire floors, and the temperature was maintained at 30, 28, and 25°C for the first, second, and subsequent weeks, respectively. Animals were provided free access to the designated diets in plastic troughs and de-ionized water in stainless-steel troughs. The experiment lasted for 6 weeks. Individual body weights and cage feed intakes were measured weekly. Daily observations were made to record general health, clinical symptoms of Se-deficiency diseases, and mortality.

**Table 1:** Composition of the basal diet (as fed)*^a^*.

**Ingredients**	**Content, g/kg**
Corn	789.0
Roasted soybean	150.0
CaCO_3_	10.0
CaHPO_4_	21.0
Salt	3.0
Choline	2.0
Trace mineral premix*^b^*	5.0
Vitamin premix*^c^*	0.5
Amino acid premix*^d^*	19.5
Total	1000.0
Nutrient composition (calculated)	
Metabolic energy, MJ/kg	12.2
Crude protein, %	19.8
Lysine, %	0.8
Methionine, %	0.5
Methionine + cysteine, %	0.7
Calcium, %	1.0
Available phosphorus, %	0.5

a *The analyzed selenium (Se) concentration in the basal diet was 10 μg/kg*.

b *Trace mineral premix provided/kg diet: FeSO_4_·7H_2_O, 379 mg; CuSO_4_·5H_2_O, 31.3 mg; ZnSO_4_·7H_2_O, 177 mg; MnSO_4_·5H_2_O, 154 mg; KI, 0.5 mg; and colistin sulfate, 40 mg*.

c *Vitamin premix provided/kg diet: retinyl acetate, 1,500 IU; cholecalciferol, 200 IU; menadione, 5 mg; thiamin, 1.8 mg; riboflavin, 3.6 mg; calcium pantothenate, 10 mg; niacin, 35 mg; pyridoxol, 3.5 mg; d-biotin, 0.15 mg; and folacin, 0.55 mg (without rac-α-tocopheryl acetate)*.

d *Amino acid premix provided per kg diet: l-lysine, 4,630 mg; dl-methionine, 3,160 mg; l-threonine, 2,010 mg; l-tryptophan, 356 mg; isoleucine, 2,020 mg; valine, 1,610 mg; phenylalanine, 2,690 mg; arginine, 2,570 mg; and glycine, 900 mg*.

### Sample Collection and Preparation

At weeks 2 and 4 of the study, chicks (*n* = 7/group) were killed by decapitation to collect blood and liver samples. The remaining animals were sampled at week 6. After immediately dissected on an ice-cold surface, the liver was perfused with ice-cold isotonic saline before being minced with surgical scissors. The minced samples were divided into aliquots, snap-frozen in liquid nitrogen, and stored at −80°C until use.

### Biochemical Assays and Histology

Se concentrations in liver and feed were measured using the hydride generation-atomic fluorescence spectrometer (AFS-3,200, Yongtuo Instruments, Beijing, China) (Moreno et al., 2000), against the standard reference of Se [GBW (E) 080441, National Research Center for Certified Reference Materials, Beijing, China]. Total GPX activity, SOD activity, and malondialdehyde (MDA) content were measured using the respective kits (Nanjing Jiancheng Bioengineering Institute, Nanjing, China). Concentrations of protein were determined using the Bradford method (Huang et al., 2015).

Histological changes due to Se and/or VE deficiency were examined by a board-certified veterinary pathologist after sections of the fixed liver were embedded in paraffin, sectioned at 6 mm, and stained with H&E.

### Real-Time Quantitative PCR Analysis of Selenoprotein mRNA Levels

Literature search allowed us to target 25 selenoprotein genes in chicks (Huang et al., 2015; Li et al., 2018). To determine the effects of dietary Se and VE on the mRNA expression of these genes, we isolated total mRNA from the liver (50–100 mg tissue) of five most representative chicks from each group. The RNA sample preparation, real-time quantitative PCR procedure, and the relative mRNA abundance qualification were the same as previously described by our group (Chen et al., 2019). Primers (Supplementary Table 1) for the 25 selenoprotein genes, 6 signal pathway genes, and 2 reference genes were as follows: β-actin gene (*ActB*) and glyceraldehyde 3-phosphate dehydrogenase gene (*Gapdh*) were designed using Primer Express 3.0 (Applied Biosystems, Foster City, CA, USA).

### Western-Blot Analyses

Tissues were homogenized with the cell lysis buffer for Western and IP (Catalog no. P0013, Beyotime Institute of Biotechnology) and centrifuged at 12,000 × *g* for 10 min at 4°C. The resulting supernatants of homogenates (10–40 μg protein/lane) were loaded onto an SDS-PAGE (12.5%), transferred to polyvinylidene difluoride membranes, and incubated with appropriate antibodies (Supplementary Table 2) as previously described (Zhang X. et al., 2020).

### Statistical Analysis

The results were analyzed by a one-way ANOVA using SPSS software (SPSS for Windows 13.0, Chicago, IL, USA). The data were presented as mean ± SE. The Duncan's multiple-range tests were used to evaluate the differences between treatments, and those differences were considered statistically significant when *P* < 0.05.

## Results

### Growth Performance, Liver Biochemical Measures, and Incidence of Liver Necrosis

The final body weight, overall daily gain, and average daily feed intake of chicks were additively decreased by dietary Se and VE deficiency. Feed/gain efficiency was decreased (*P* < 0.05) by dietary Se deficiency (Table 2). Dietary Se deficiency decreased (*P* < 0.05) hepatic Se concentrations at weeks 2 and 4 (Table 3). The –Se chicks had ~37% of the hepatic Se concentration of the +Se chicks at two time points. The –Se chicks had ~50% and ~22% of the GPX activities of the +Se chicks at weeks 2 and 4, respectively. Moreover, Se deficiency elevated the MDA content and decreased SOD in the liver at two time points. In the +SE–VE group, only the MDA content at week 4 was significantly higher than for the +SE+VE group. Although there was no incidence of liver necrosis, ED, and pancreatic atrophy (data not shown) in any of the groups by week 2, the symptoms of liver necrosis (Supplementary Figure 1) were first found in one chick at day 16 in the –SE–VE group. Thereafter (from weeks 3 to 6), 11 chicks showed symptoms of liver necrosis in the –SE–VE group. Meanwhile, 5 chicks showed symptoms of liver necrosis in the –SE+VE group throughout the experimental period. Typical signs of hepatic necrosis in the –SE chicks showed varying degrees of swelling, purple, red or yellow of color, fragile, and pinpoint bleeding in the liver. Likewise, dietary Se deficiency also induced typical signs of necrosis and vacuolar degeneration in the liver (Supplementary Figure 1) of chicks at week 3 and thereafter.

**Table 2:** Effects of dietary Se and vitamin E (VE) on growth performance in chicks^1^.

**Se, mg/kg**	**0**	**0**	**0.3**	**0.3**
**Vitamin E, mg/kg**	**0**	**50**	**0**	**50**
Initial weight, g	46.3 ± 2.1 (40)	45.7 ± 2.6 (40)	46.2 ± 3.1 (40)	46.3 ± 2.5 (40)
Final weight, g	1082.4 ± 36.7^d^(6)	1301.9 ± 73.4^c^(18)	1837.4 ± 73.2 ^b^(26)	1973.4 ± 79.8^a^(26)
ADG, g/d	24.7 ± 3.2^d^(–^2^)	29.4 ± 2.1^c^(–)	42.6 ± 2.2^b^(–)	45.9 ± 3.3^a^(–)
ADFI, g/d	66.7 ± 3.2^c^(–)	76.5 ± 5.2^b^(–)	88.4 ± 4.3^a^(–)	91.9 ± 4.7^a^(–)
F/D	2.7	2.6	2.1	2.0

1 *Values are means ± SE. Means within each item differ (P < 0.05) without sharing a common superscript letter (a, b, c, d)*.

2 *n = 6–40*.

*ADG, average daily; ADFI, average daily feed intake; F/G, feed-to-gain ratio*.

**Table 3:** Effects of dietary Se and VE concentrations on Se concentrations of tissues and biochemical indicators in chick liver.

**Se, mg/kg**	**0**	**0**	**0.3**	**0.3**
**Vitamin E, mg/kg**	**0**	**50**	**0**	**50**
Se concentrations, ng/g tissue				
Week 2	172.0 ± 17.2^b^	169.7 ± 16.2^b^	423.0 ± 41.0^a^	435.7 ± 34.6^a^
Week 4	144.7 ± 17.6^b^	143.3 ± 11.5^b^	424.3 ± 38.7^a^	422.7 ± 39.5^a^
GPX activity, unit/mg protein^2^				
Week 2	4.9 ± 0.5^b^	5.0 ± 0.6^b^	10.4 ± 0.9^a^	11.1 ± 1.2^a^
Week 4	2.2 ± 0.3^b^	2.5 ± 0.4^b^	11.4 ± 0.7^a^	9.8 ± 0.8^a^
SOD activity, unit/mg protein				
Week 2	7.3 ± 0.8^b^	8.5 ± 0.8^b^	16.8 ± 1.5^a^	17.9 ± 1.4^a^
Week 4	5.3 ± 0.7^b^	5.0 ± 0.6^b^	21.7 ± 1.1^a^	22.2 ± 0.7^a^
MDA content, unit/mg protein				
Week 2	8.6 ± 0.9^a^	6.2 ± 0.7^b^	4.7 ± 0.4^c^	4.5 ± 0.5^c^
Week 4	10.2 ± 1.3^a^	8.7 ± 0.9^b^	5.9 ± 0.5^c^	3.8 ± 0.3^d^

1 *Values are means ± SE, n = 5. Means in the row without a common letter differ, P < 0.05*.

2 *1 μmol glutathione oxidized/min at 37°C*.

### Real-Time Quantitative PCR Analysis of Selenoprotein mRNA Levels

**Figure 1:**
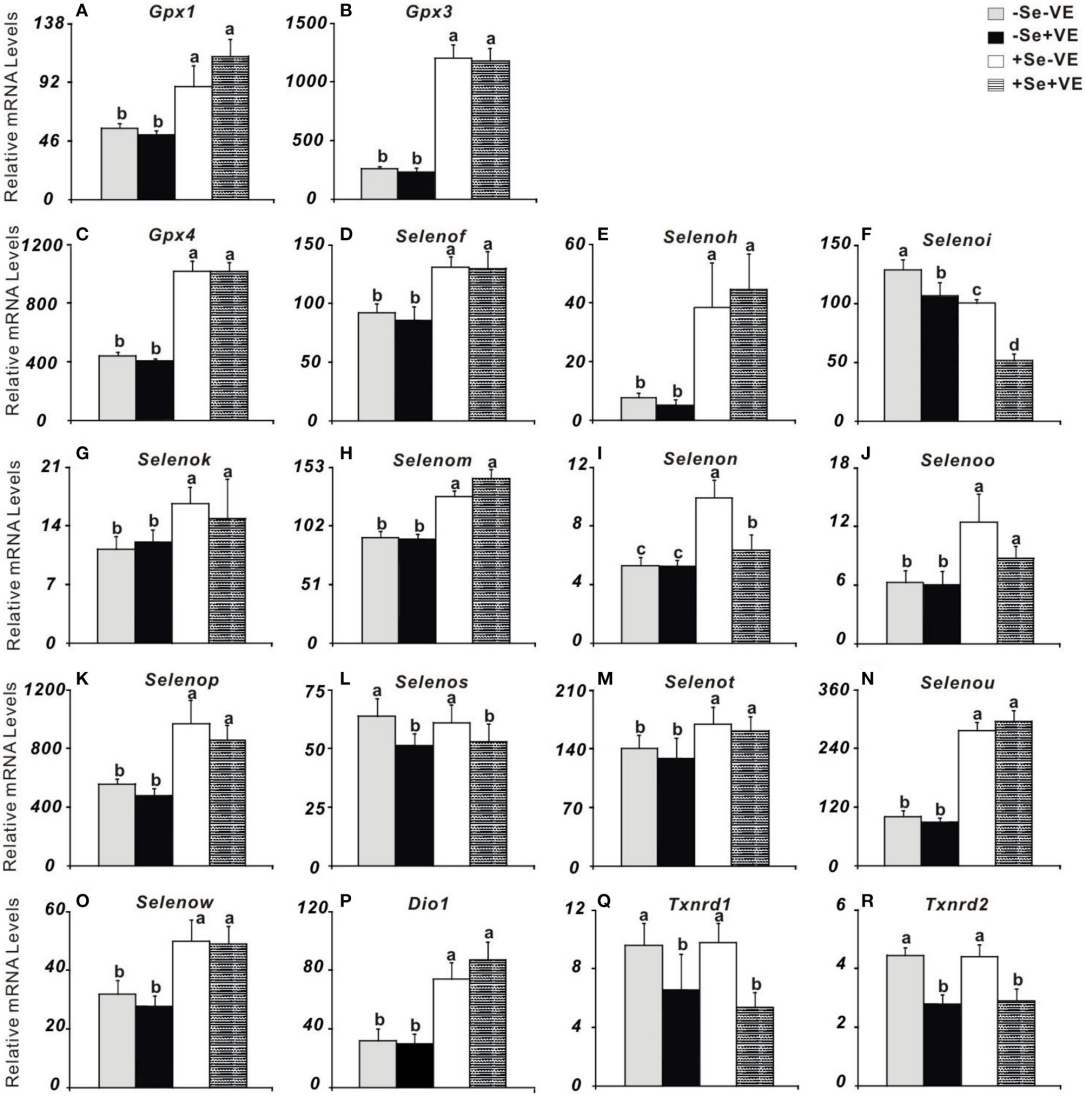
Effects of dietary selenium (Se) and vitamin E (VE) concentrations on relative mRNA abundance of **(A)** Gpx1, **(B)** Gpx3, **(C)** Gpx4, **(D)** Selenof, **(E)** Selenoh, **(F)** Selenoi, **(G)** Selenok, **(H)** Selenom, **(I)** Selenon, **(J)** Selenoo, **(K)** Selenop, **(L)** Selenos, **(M)** Selenot, **(N)** Selenou, **(O)** Selenow, **(P)** Dio1, **(Q)** Txnrd1, and **(R)** Txnrd2 in muscle of chicks at wk 2. Data are means ± SE, n = 5. Values within a given gene differ (P < 0.05) without sharing a common superscript letter.

Dietary Se exerted the main effect (*P* < 0.05) on 15 out of 25 selenoprotein genes assayed in the liver at week 2, whereas there was a main effect of dietary VE (*P* < 0.05) or an interaction between these two nutrients (*P* < 0.01) on 5 selenoprotein genes (Figure 1). As shown in Figure 1, chicks fed with the two Se-deficient diets displayed lower (*P* < 0.05) hepatic mRNA levels of *Gpx1, Gpx3, Gpx4, Selenof, Selenoh, Selenok, Selenom, Selenon, Selenoo, Selenop, Selenot, Selenou, Selenow*, and *Dio1* than those fed with the two Se-supplemented diets. Conversely, liver mRNA levels of *Selenoi* were actually greater (*P* < 0.05) in the chicks fed with the Se-deficient diets than those fed with the Se-supplemented diets. Liver mRNA levels of *Selenon* were decreased (*P* < 0.05) in the Se-adequate chicks by dietary VE supplementation. Notably, chicks fed with the two VE-supplemented diets had lower (*P* < 0.05) liver mRNA levels of *Selenoi, Selenos, Txnrd1*, and *Txnrd2* than those fed with the VE-deficient diets at both levels of dietary Se concentrations. Neither dietary Se nor VE affected liver mRNA levels of *Gpx2, Selenopb, Msrb1, Txnrd3, Dio2*, or *Dio3*.

### Abundance of Selenoproteins and Related Histopathological Condition of Liver

**Figure 2:**
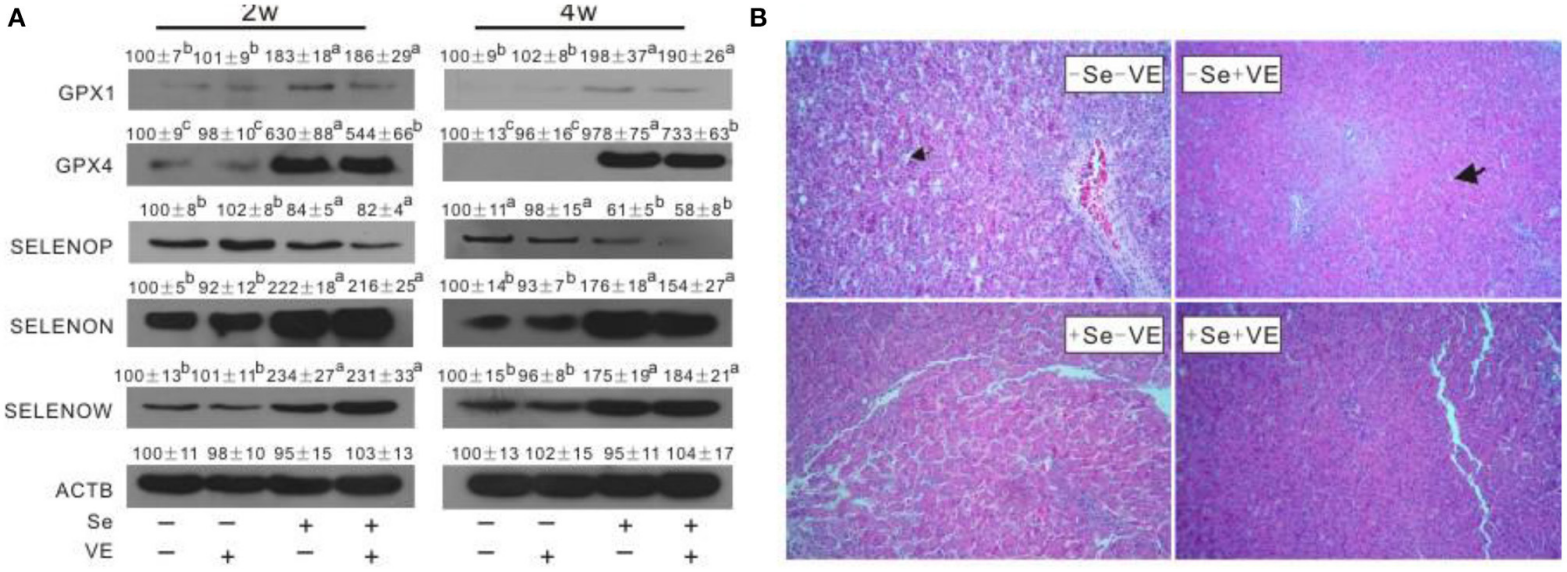
Effects of dietary Se and VE on hepatic **(A)** protein levels of GPX1, GPX4, SELENOP, SELENON, and SELENOW in chick at weeks 2 and 4 and **(B)** histopathological changes in chick at week 4. The image **(B)** is representative of five sets of data. Original magnification 400×. Data are means ± SE, *n* = 3. Values within a given gene differ (*P* < 0.05) without sharing a common superscript letter.

The abundance of proteins, such as GPX1, GPX4, SELENOP, SELENOW, and SELENON, were affected (*P* < 0.05) in the liver by dietary Se concentrations (Figure 2A). Dietary Se deficiency increased (*P* < 0.05) SELENOP, but decreased (*P* < 0.05) GPX1,GPX4, SELENOW, and SELENON in the liver of chicks at weeks 2 and 4. Notably, chicks fed with the VE-supplemented diet had lower (*P* < 0.05) impact on the abundance of GPX4 in the liver in +Se chicks at two time points. Neither dietary Se nor VE affected GPX3 and MSRB1 in the liver at two time points. We observed liver tissues stained by H&E at 4 weeks (Figure 2B). The liver tissues in the +Se chicks displayed normal morphologies. Compared with the +Se chicks, the liver parenchyma was partially loose and accompanied by lymphocytic infiltration in the –SE chicks. Furthermore, the vascular and bile duct walls in the portal area were incomplete, and the hepatic sinusoidal space had increased and contained abundant red blood cells.

### Liver Necroptosis Related Gene Expression and Protein Production

**Figure 3:**
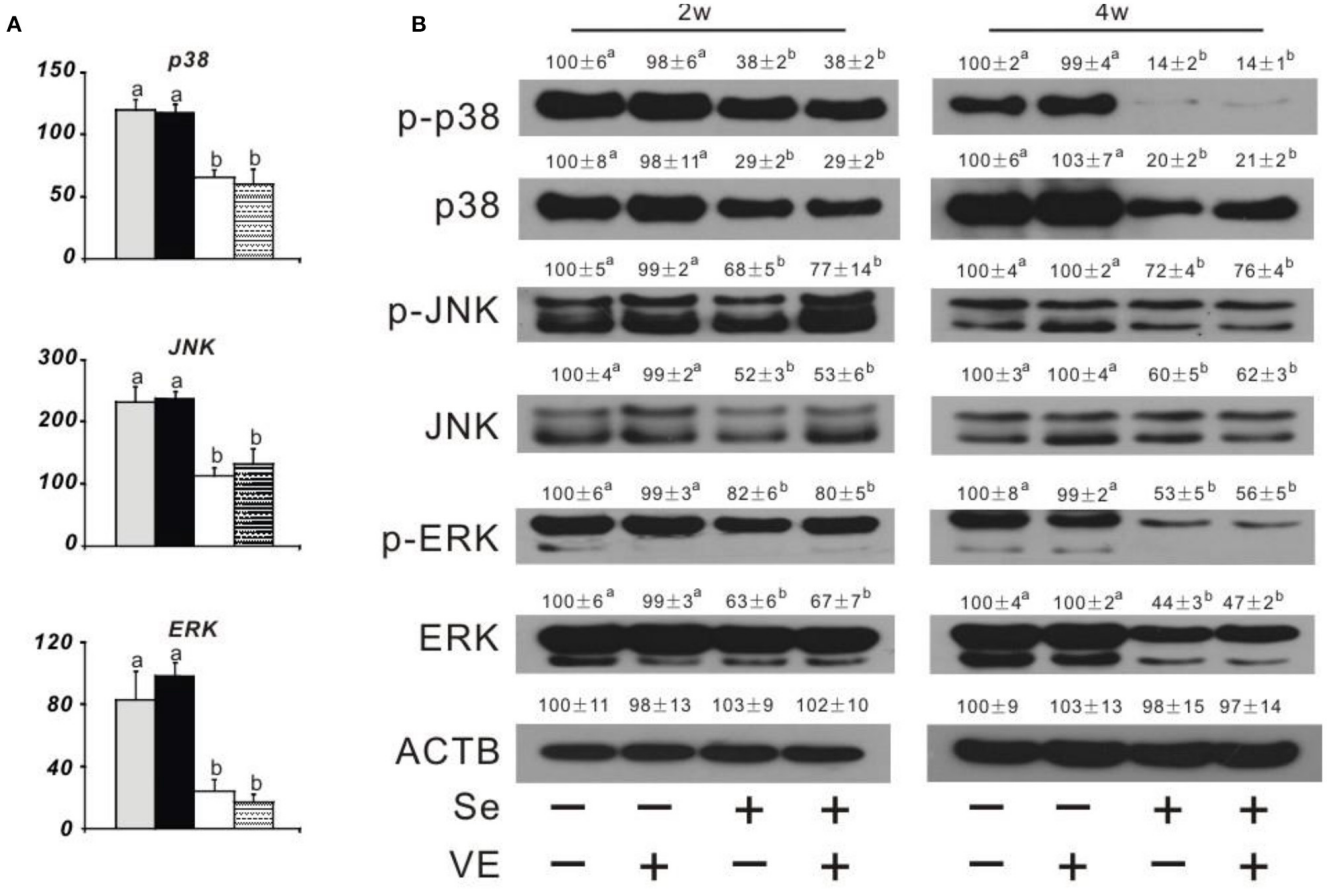
Effects of dietary Se and VE on hepatic **(A)** relative mRNA abundance of p38, JNK, and *ERK* in chick at week 4 and **(B)** protein levels of phospho-p38 mitogen-activated protein kinase (*p*-p38), p38 mitogen-activated protein kinase (p38), phospho-c-Jun N-terminal kinase (p-JNK), c-Jun N-terminal kinase (JNK), phospho-mitogen-activated protein kinase (p-ERK), extracellular signal-regulated kinase (ERK), and β-actin gene (ACTB) in chick at weeks 2 and 4. Data are means ± SE, *n* = 5 **(A)** or 3 **(B)**. Values within a given gene differ (*P* < 0.05) without sharing a common superscript letter.

**Figure 4:**
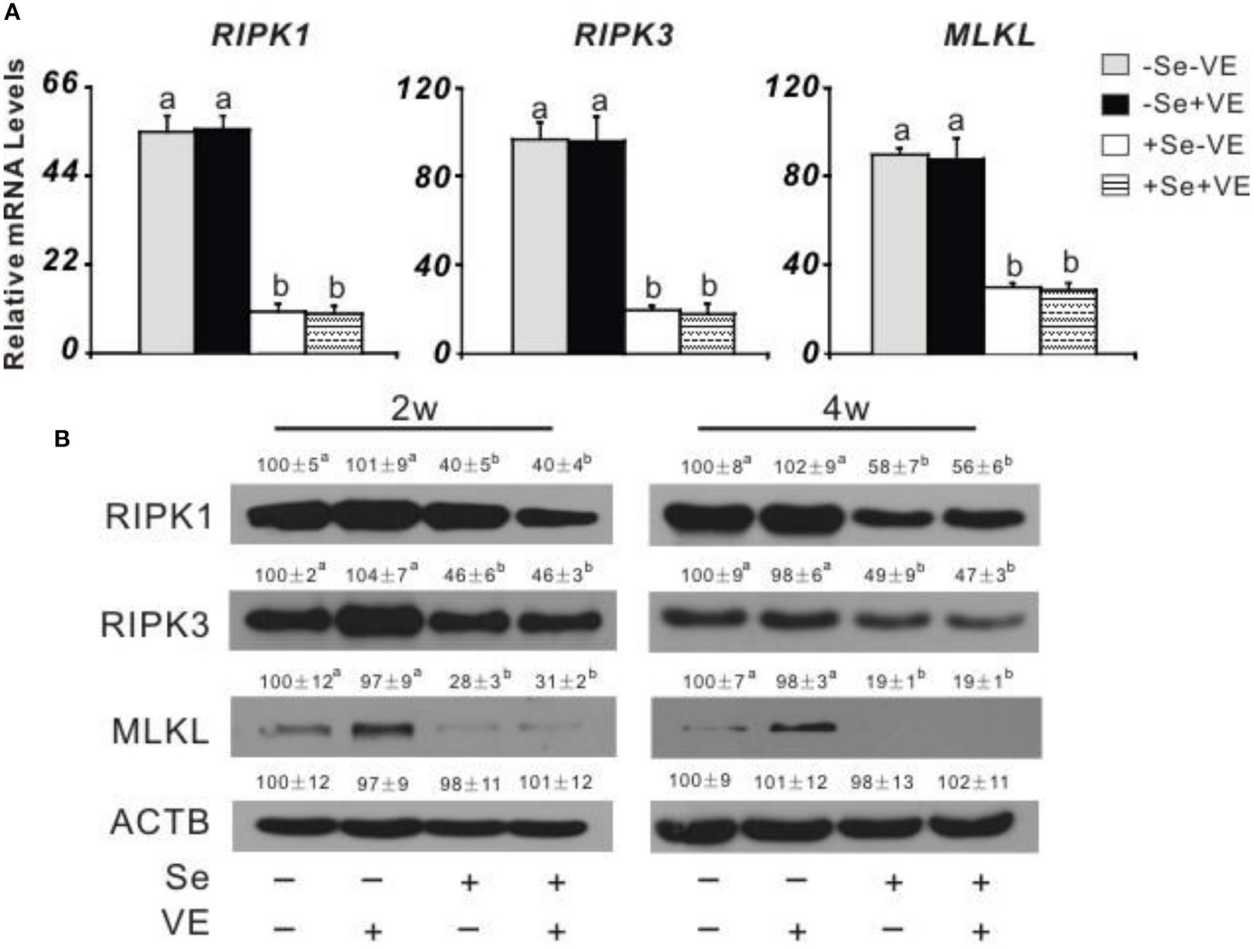
Effects of dietary Se and VE on hepatic **(A)** relative mRNA abundance of receptor-interacting serine-threonine kinase 1 (*RIPK1*), receptor-interacting serine-threonine kinase 3 (*RIPK3*), and mixed lineage kinase domain-like (*MLKL*) in chick at week 4 and **(B)** protein levels of RIPK1, RIPK3, MLKL, and ACTB in chick at weeks 2 and 4. Data are means ± SE, *n* = 5 **(A)** or 3 **(B)**. Values within a given gene differ (*P* < 0.05) without sharing a common superscript letter.

Dietary Se deficiency increased (*P* < 0.05) (Figure 3A) relative gene expression of p38, JNK, and ERK in the liver of chicks at week 4 and (Figure 3B) protein levels of p38, p-p38, JNK, p-JNK, ERK, and p-ERK in the liver of chicks at weeks 2 and 4. Meanwhile, dietary Se deficiency increased (*P* < 0.05) (Figure 4A) relative gene expression of *RIPK1, RIPK3*, and *MLKL* in the liver of chicks at week 4 and (Figure 4B) protein levels of RIPK1, RIPK3, and MLKL in the liver of chicks at weeks 2 and 4. However, liver necroptosis-related gene expression and protein production were not affected at two time points in chicks fed with the VE-supplemented diet.

## Discussion

The antioxidant effect of Se is mainly realized through selenoprotein, and an appropriate amount of Se can reduce the concentration of free radicals and thus protect organisms from lipid peroxidation. Furthermore, VE as a non-specific biological antioxidant could compensate and coordinate with Se in protecting cell membranes from oxidative damage (Huang et al., 2011). Previous studies have found that the lack of Se and VE in diet could lead to liver necrosis in chicks. In this study, we successfully replicated liver necrosis in chicks by feeding the Se-deficient diets. The signs of liver necrosis were observed, namely, varying degrees of swelling, purple, red or yellow color, fragility, and pinpoint bleeding in the liver (Sun et al., 2016). No incidence of liver necrosis was observed at any time point in the +Se chicks. Dietary Se concentrations exerted the intended impacts on the Se status of chicks, as shown by the Se concentrations in liver and GPX activities (Taylor et al., 2018). The lack of Se and VE in chicks leads to hepatic necrosis and increased peroxidation (Guillermo et al., 2018). Lipid peroxidation may cause membrane destruction and cell death, thereby triggering the process of necrosis (Burk et al., 1980; Zhang Y. et al., 2020). Although the mechanistic basis of mitochondrial injury may vary in different settings, mitochondrial permeability and dysfunction are common events that lead to cell apoptosis and necrosis (Guicciardi et al., 2013). Generally, apoptosis and necrosis are considered to be separate entities, but an alternate view is emerging that both are frequently a result of the same initiating factors and signaling pathways. Furthermore, apoptosis and necrosis in their pure form may represent extremes of a continuum of cell death rather than being separate entities (Zhang Y. et al., 2020). However, the protection of VE against the incidence of liver necrosis in the Se-deficient chicks was seemingly independent of muscle GPX and SOD activities. It is unclear whether these two biomarkers (i.e., GPX and SOD) were not sufficiently sensitive to reflect the effects of the relatively low dose of VE (50 mg/kg) supplementation or if VE protection was mediated by other mechanisms.

The available information on selenoprotein genes of novel chicken enabled us to design primers for qPCR to complete the systematic analysis of the effects of dietary Se on 25 selenoprotein genes expression profiles of chicken (Li et al., 2018; Sun et al., 2018). Dietary Se deficiency decreased (*P* < 0.05) the mRNA levels of *Gpx1, Gpx3, Gpx4, Selenof, Selenoh, Selenok, Selenom, Selenoo, Selenop, Selenou*, and *Selenow* in liver at week 2. And six common genes (*Gpx1, Gpx4, Selenok, Selenoo, Selenop*, and *Selenow*) were downregulated by dietary Se deficiency in liver and muscle at weeks 2 and 6 (Huang et al., 2015; Sun et al., 2018). In addition to the analogous response of these selenoprotein genes in other animals (Huang et al., 2015; Sunde et al., 2016; Sun et al., 2018; Chen et al., 2019; Zhang Y. et al., 2020), these genes can be used as indicators of Se status of the body in chicks. In addition, further functional assessments should be conducted to determine whether the downregulation of these genes is associated with hepatic necrosis. Moreover, the responses of liver *Selenoi* and muscle *Selenon* mRNA levels were different between the time points (weeks 2 and 6). SELENOI (encoded by *Selenoi*) is an evolved selenoprotein specific to vertebrates and is essential for murine embryogenesis (Avery et al., 2020). However, this membrane selenoprotein has no known function. Recent studies have identified selenoprotein N (encoded by *Selenon*) as an important protein that protects cells against oxidative stress and redox-related calcium homeostasis (Shin et al., 2019). SELENON is an endoplasmic reticulum calcium sensor that links luminal calcium levels to a redox activity (Pozzer et al., 2019).

At week 6, dietary VE deficiency upregulated hepatic *Selenoi, Selenos, Txnrd1*, and *Txnrd2* mRNA levels at week 2. While three hepatic selenoprotein genes (i.e., *Selenoi, Txnrd1*, and *Txnrd2*) show the same trend at two time points, the expression of *Gpx1* and *Selenos* was affected by VE supplementation at different time points in the liver. Overall, the selenoprotein gene mRNA levels have an inverse relationship with VE status, and further protein assays should be conducted in the future. In the liver, three additional selenoprotein genes (i.e., *Selenon, Selenot*, and *Dio1*) were downregulated by dietary Se deficiency at week 2. However, among the 25 genes assayed, only *Selenoi* mRNA level was decreased by dietary Se supplementation. Additionally, liver mRNA levels of *Selenon* were decreased in Se-adequate chicks by dietary VE supplementation. The gene code for protein is associated with neuroprotective function and cytosolic calcium regulation (*Selenom*) (Huang et al., 2016). In conclusion, responses of selenogenome expression in the liver of chicks to dietary Se and VE deficiencies differed between the time period before and the time period after the onset of liver necrosis. These differences may offer us a new clue on the role of selenoproteins in the pathogenesis of liver necrosis.

Although Se deficiency affects gene expression in multiple tissues (Liu et al., 2012; Yao et al., 2013; Sun et al., 2018), the selenogenome that are regulated is diverse, tissue-specific, and depends on their functions in the tissue (Joseph and Peter, 2018). The liver, in addition to its target organ of liver necrosis, has many important functions in performing and regulating diverse metabolic processes (Zhang Y. et al., 2020). In order to better understand the impact of selenoprotein influenced by liver necrosis, this study revealed novel regulations of protein production of GPX1, GPX3, GPX4, SELENOP, SELENON, SELENOW, and MsrB1 in the liver of chicks by dietary Se and/or VE at weeks 2 and 4. The tissue protein levels of these seven selenoproteins responded to dietary Se concentrations in three shapes. First, the levels of four selenoproteins (i.e., GPX1, GPX4, SELENOW, and SELENON) in the liver showed similar downregulations by dietary Se deficiency at two time points. At present, the functions of GPX1, GPX4, SELENOW, and SELENON are key redox enzymes in oxidative defense. Second, the abundance of SELENOP in the liver was elevated by dietary Se deficiency at two time points. SELENOP accounts for >50% of the total plasma Se content and exists in the blood as at least two isoforms with molecular weights of 50 and 60 kDa, respectively (Huang et al., 2015). Studies on transgenic mice lacking the *Selenop* gene have shown that this protein has an important function in delivering Se from the liver to extra-hepatic tissues, such as the brain and testis (Evangelos et al., 2018; Saito, 2019). The opposite influence of SELENOP between mRNA and protein by Se supplementation is hard to explain. Finally, GPX3 and MsrB1 showed no change of protein levels in the liver at two time points. GPX3, also known as plasma or extracellular GPX, is a selenoprotein mainly secreted by the proximal tubule cells of the kidney. MsrB1 (also called selenoprotein R) was first identified as a selenoprotein through bioinformatics methods (Lee et al., 2020). MsrB1 deficiency exacerbates the hepatotoxicity caused by acetaminophen *via* increased oxidative damage (Kim et al., 2017). Among the seven selenoproteins assayed, only the GPX4 level was decreased by VE supplementation in the liver of +Se chicks. Downregulation of muscle GPX4 protein by VE supplementation in the +Se chicks may be partially explained by a mutually sparing mechanism due to their overlap function of protecting against lipid peroxidation. As mentioned above, tissue *Gpx4* mRNA, GPX4 activity, and GPX4 protein levels were affected by dietary Se in chicks. Another unique feature of selenoprotein abundance in broilers is that GPX4 protein levels were ~83–99% lower in the liver of –Se chicks, compared with those of +Se chicks. GPX4 is the only GPX that accepts phospholipid hydroperoxidase as an oxidation substrate in membranes, as well as uses protein-thiol groups as a reduction substrate in the absence of GSH (Ding et al., 2021). Since early embryonic lethality of GPX4-knockout mice limits further *in vivo* studies on the molecular and cellular mechanisms of GPX4, we have speculated that avian GPX4 could function as a redox sensor (Huang et al., 2015). In this study, the incidences of hepatic necrosis in chicks were completely prevented by dietary Se supplementation, and the necrotic mechanism induced by Se deficiency was still unknown. In this study, we identified appropriate antibodies from multiple sources to assay several major cell signaling pathway proteins that may be related to RIPK1/RIPK3/MLKL and MAPK pathway (see below). Compared with those of the Se-adequate chick, the Se-deficient chick had greater hepatic protein levels of RIPK1, RIPK3, MLKL, p38, p-p38, JNK, p-JNK, ERK, and p-ERK. Previous studies have shown that cell necroptosis was determined by increasing the levels of RIPK1, RIPK3, and MLKL and decreasing the expression of caspase 8 (Guida et al., 2017; Cui et al., 2020). Activation of RIPK1 triggers mitochondrial damage, which in turn promotes higher levels of oxidative stress and ultimately leads to cell death. It is suggested that the apoptotic pathway may partially develop into necrosis (Yan et al., 2018). Se deficiency leads to MLKL-dependent necroptosis and mitochondrial apoptosis in Se-deficient animal models (Li et al., 2019; Wang H. et al., 2020). The ubiquitination of RIPK1, RIPK3, and recruitment of the downstream mediator MLKL resulted in a necrotic-like cell death process called necroptosis (Shi et al., 2019). The previous study demonstrated that arbutin protects retinal ganglion cells against oxidative injury induced by H_2_O_2_ *via* inhibiting the p38MAPK and MEK/ERK signaling pathways (Zhao et al., 2019). Induction of phosphorylated p38, JNK, and ERK, the active forms of the protein kinases, occurs due to Se deficiency in chicks. Compared with the wild type, the two knockout mice (i.e., SOD^−/−^and GPX1^−/−^) had higher levels of phospho-p38 MAPK (Wang et al., 2011). Meanwhile, there were significantly increased levels of phosphorylated ERK1/2 in the GPX4^+/−^mice. JNK is a group of mitogen-activated protein kinases activated by cytokines or environmental stress that participates in a variety of signaling pathways, including apoptosis pathways. Interestingly, the low activity of GPX1 (4% of the adequate level) in Se-deficient mice was sufficient to attenuate low-dose pro-oxidant-mediated JNK phosphorylation at Thr-183/Tyr-185 (Huang et al., 2018). Overall, these signaling proteins were first reported in hepatic necrosis induced by Se and/or VE deficiency, and the postulated mechanism was that the RIPK1/RIPK3/MLKL and MAPK signaling pathways were activated by Se deficiency to protect the liver from necrosis.

## Conclusion

**Figure 5:**
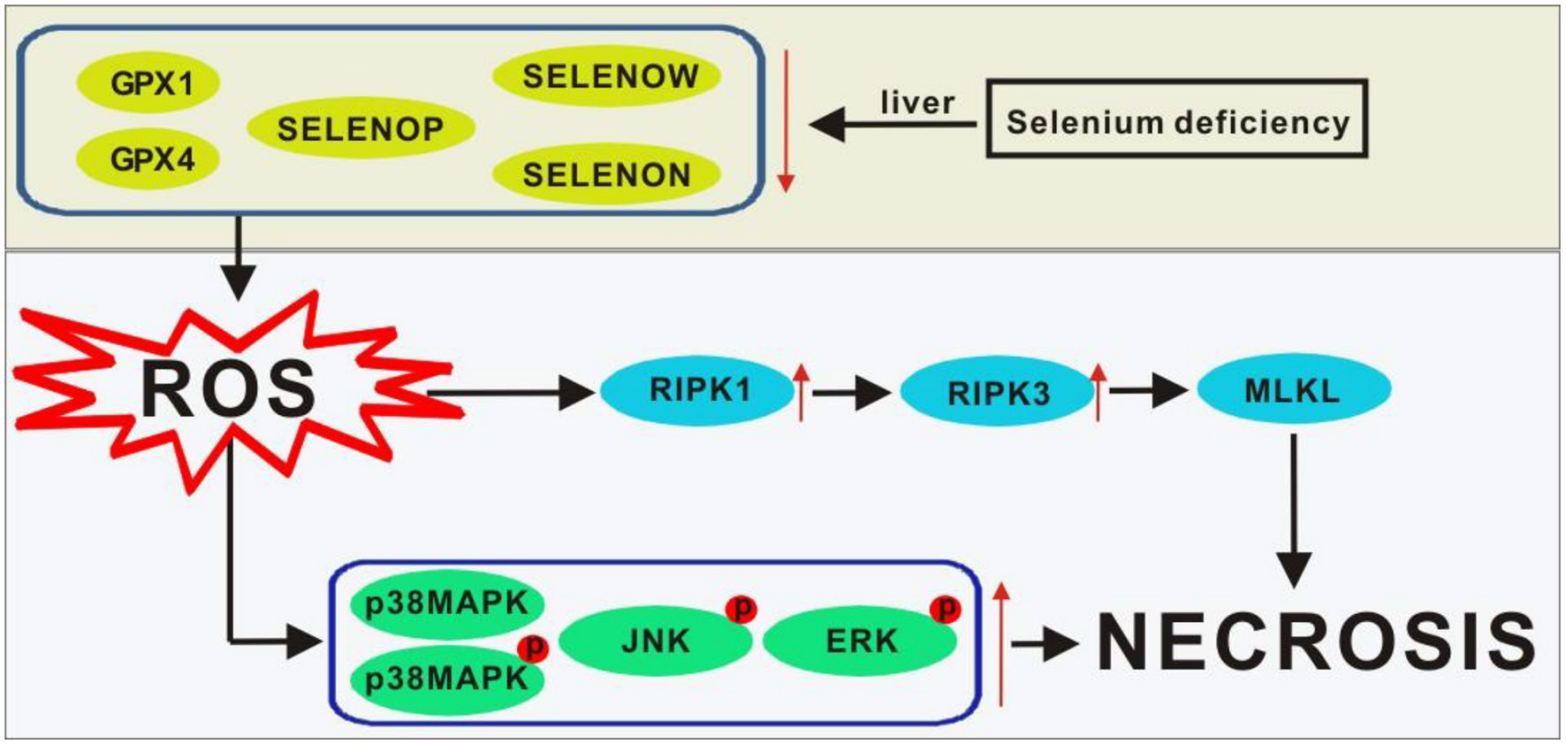
Scheme of postulated regulatory pathways and mechanisms for the liver necrosis induced by Se deficiency. Lines ending with arrows, activation or increase; ↑, activation or increase; ↓, inhibition or decrease. GPX1, glutathione peroxidase-1; GPX4, glutathione peroxidase-4; MLKL, mixed lineage kinase domain-like; p38MAPK, p38 mitogen-activated protein kinase; *p*-p38MAPK, phosphorylated p38MAPK; p-JNK, phosphorylated JNK; p-ERK, phosphorylated ERK; ROS, reactive oxygen species; RIPK1, receptor-interacting serine-threonine kinase 1; RIPK3, receptor-interacting serine -threonine kinase 3; Se, selenium; SELENON, selenoprotein N; SELENOP, selenoprotein P; SELENOW selenoprotein W.

In summary, our data revealed the differential regulation of dietary Se deficiency on 25 selenoprotein genes, 7 key selenoproteins, and the RIPK1/RIPK3/MLKL and MAPK signaling pathway proteins in chicks and provided new molecular clues for understanding the pathogenesis of liver necrosis. The protective effects of Se are probably exerted *via* peroxide scavenging, controlling oxidative stress, and regulating subsequent stress signaling (Miyata et al., 2020). As shown in Figure 5, the main stress signaling proteins were activated, including p38 MAPK, JNK, and ERK. Additionally, the RIPK1/RIPK3/MLKL signaling pathway was involved in mediating the protection conferred by the five selenoproteins against the onset of liver necrosis (Wang H. et al., 2020).

## Data Availability Statement

The original contributions presented in the study are included in the article/Supplementary Material, further inquiries can be directed to the corresponding authors.

## Ethics Statement

The animal study was reviewed and approved by China Agricultural University.

## Author Contributions

J-QH provided ideas for the experiment. TL and JZ completed the experiment and wrote the manuscript. Z-WZ and P-JW completed the parts of the figures. All authors contributed to the article and approved the submitted version.

## Conflict of Interest

The authors declare that the research was conducted in the absence of any commercial or financial relationships that could be construed as a potential conflict of interest.

## Publisher's Note

All claims expressed in this article are solely those of the authors and do not necessarily represent those of their affiliated organizations, or those of the publisher, the editors and the reviewers. Any product that may be evaluated in this article, or claim that may be made by its manufacturer, is not guaranteed or endorsed by the publisher.
