# Supplementary material for: Selenoproteins Protect Against Avian Liver Necrosis by Metabolizing Peroxides and Regulating Receptor Interacting Serine Threonine Kinase 1/Receptor Interacting Serine Threonine Kinase 3/Mixed Lineage Kinase Domain-Like and Mitogen-Activated Protein Kinase Signaling
Source: Front Physiol. 2021 Aug 12;12:696256. doi: 10.3389/fphys.2021.696256 (PMC8397447; doi:10.3389/fphys.2021.696256)
Supplement: Supplementary file 1 [file Data_Sheet_1.docx]

**Supple figure 1** The clinical symptoms of liver necrosis in chicks at week 4. Typical signs of hepatic necrosis in the –SE chicks showed varying degrees of swelling, purple, red or yellow of color, fragile and needle-like bleeding in liver.

**
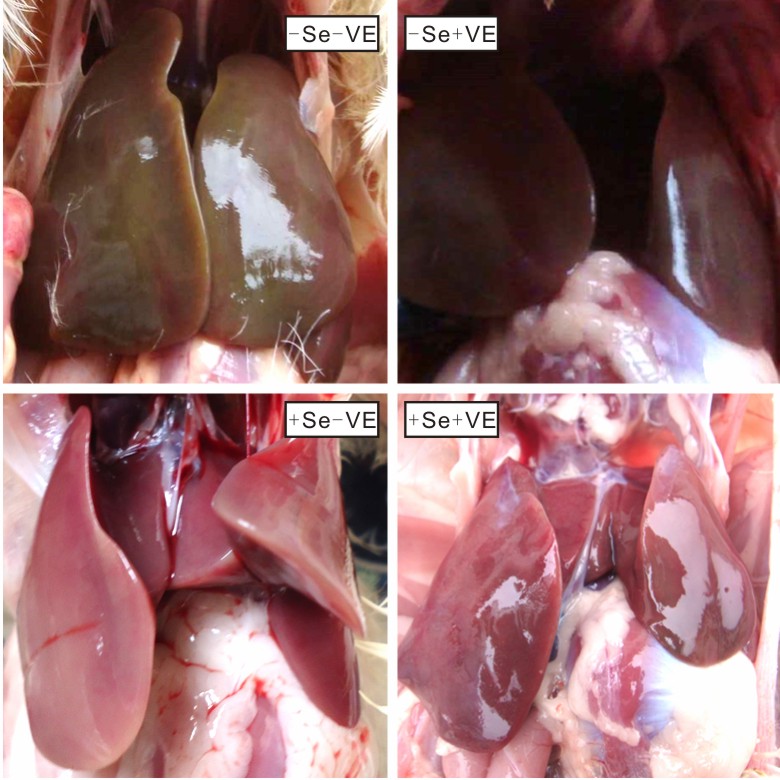
**

**Supple figure 2** Effects of dietary Se and vitamin E on hepatic protein levels of GPX3 and MSRB1 in chick at weeks 2 and 4. Data are means ± SE, n = 3.Values within a given gene differ (P < 0.05) without sharing a common superscript letter.

**
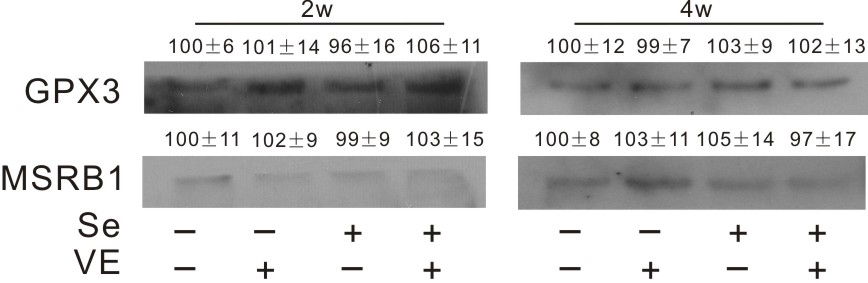
**

**Supplemental table 1** Gene-special primers used in the qPCR ^1^.

| **Gene** | **Forward primer** | **Reverse primer** |
| --- | --- | --- |
| *Gpx1* | ACGGCGCATCTTCCAAAG | TGTTCCCCCAACCATTTCTC |
| *Gpx2* | ATCGCCAAGTCCTTCTACGA | ACGTTCTCGATGAGGACCAC |
| *Gpx3* | CCTGCAGTACCTCGAACTGA | CTTCAGTGCAGGGAG GATCT |
| *Gpx4* | CTTCGTCTGCATCATCACCAA | TCGACGAGCTGAGTGTAATTCAC |
| *Selenof* | ACTTGGCTTCTCCAGTAACTTGCT | GCCTACAGAATGGATCCAACTGA |
| *Selenoh* | CATCGAGCACTGCCGTAG | GACACCTCGAAGCTGTTCCT |
| *Selenoi* | TGCCAGCCTCTGAACTGGAT | TGCAAACCCAGACATCACCAT |
| *Selenok* | GAAGAGGGCCTCCAGGAAAT | CAGCCATTGGTGGTGGACTAG |
| *Selenom* | AAGAAGGACCACCCAGACCT | GCTGTCCTGTCTCCCTCATC |
| *Selenon* | CAGGATCCATGCTGAGTTCCA | GAGAGGACGATGTAACCCGTAAAC |
| *Selenoo* | CCAGCGTTAACCGGAATGAT | ATGCGCCTCCTGGATTTCT |
| *Selenop* | CCAAGTGGTCAGCATTCACATC | ATGACGACCACCCTCACGAT |
| *Selenopb* | AGGCCAACAGTACCATGGAG | GTGGTGAGGATGGAGATGGT |
| *Selenos* | CCGACATGGTGGTAAGAAGACA | GCTTGTGCATTCAACTCCTCTTG |
| *Selenot* | AGGAG TACAT GCGGG TCATC A | GACAG ACAGG AAGGA TGCTA TGTG |
| *Selenou* | GATGCTTTCAGGCTTCTTCC | CTGTCTTCCTGCTCCAATCA |
| *Selenow* | TGGTGTGGGTCTGCTTTACG | CCAAAGCTGGAAGGTGCAA |
| *Msrb1* | TGGCAAGTGTGGCAATGG | GAATTTGAGCGAGCTGCTGAAT |
| *Txnrd1* | TACGCCTCTGGGAAATTCGT | CTTGCAAGGCTTGTCCCAGTA |
| *Txnrd2* | GCTCTTAAAGATGCCCAGCACTAC | GAACAGCTTGAGCCATCACAGA |
| *Txnrd3* | CCTGGCAAAACGCTAGTTGT G | CGCACCATTACTGTGACATCTAGAC |
| *Dio1* | GCGCTATACCACAGGCAGTA | GGTCTTGCAAATGTCACCAC |
| *Dio2* | ATTTGCTGATCACGCTTCAG | GCTCAGAAACAGCACCATGT |
| *Dio3* | CTGTGCATTCGCAAGAAGAT | GCCGACTTGAAGAAGTCCAG |
| *RIPK1* | AAGGGCGTTTCATCCTGGAG | CGGCAGGTCTCTTCTTTGGT |
| *RIPK3* | CCCATGGACAGGGAATGGAA | CACAAGTCTCTGGTAGCGG |
| *MLKL* | CCATGGGTGGTTCCTCCTTC | TGGATCTTCCGCACCTTAGC |
| *p38* | GCGAGTCCCTAATGCCTACG | ACAACTGTTGAGCCACACTCA |
| *JNK* | TGACCGAGTGAGGAGACGAT | ACTGTATCGAACGCAGCACA |
| *ERK* | AGAATCTCACAGCGTCTCGC | GGTGTGATTCATCAGCATCTTCA |
| *ActB* | ACCTGAGCGCAAGTACTCTGTCT | CATCGTACTCCTGCTTGCTGAT |
| *Gapdh* | GGTGCTAAGCGTGTTATCATCTCA | CATGGTTGACACCCATCACAA |

^1^*Gpx1*: Cytosolic glutathione peroxidase; *Gpx2*: Gastro-intestinal glutathione peroxidase; *Gpx3*: Plasma glutathione peroxidase; *Gpx4*: Phospholipidhydroperoxide glutathione peroxidase; *Selenof*: Selenoprotein 15; *Selenoh*: Selenoprotein H; *Selenoi*: Selenoprotein I; *Selenok*: Selenoprotein K; *Selenom*: Selenoprotein M; *Selenon*: Selenoprotein N; *Selenoo*: Selenoprotein O; *Selenop*: Selenoprotein P; *Selenopb*: Selenoprotein Pb; *Selenos*: Selenoprotein S; *Selenot*: Selenoprotein T; *Selenou*: Selenoprotein U; *Selenow*: Selenoprotein W; *Msrb1*: Selenoprotein X; *Txnrd1*, Thioredoxin reductase 1; *Txnrd2*, Thioredoxin reductase 2; *Txnrd3*, Thioredoxin reductase 3; *Dio1*, Iodothyronine deiodinases 1; *Dio2* Iodothyronine deiodinases 2; *Dio3*, iodothyronine deiodinases 3; *RIPK1*, receptor interacting serine threonine kinase 1; *RIPK3*, receptor interacting serine threonine kinase 3; *MLKL*, mixed lineage kinase domain-like; p38, p38 mitogen-activated protein kinase; *JNK*, c-Jun N-terminal kinase; *ERK*, mitogen-activated protein kinase; *ActB*, beta-actin; *Gapdh*, Glyceraldehyde 3-phosphate dehydrogenase.

**Supplemental table2** Name, type, dilution, and source of primary antibodies used for the western analyses of selenoproteins and an signaling pathway proteins in tissues of chicks fed different concentrations of dietary Se and/or vitamin E.

| Antibody^1^ | Species | Isotype | Dilution | Source |
| --- | --- | --- | --- | --- |
| GPX1 | Human | Rabbit | 1:500 | Abcam (Cambridge, MA) |
| GPX3 | Human | Rabbit | 1:500 | Abcam (Cambridge, MA) |
| GPX4 | Human | Rabbit | 1:1000 | Santa Cruz Biotechnology(Santa Cruz, CA) |
| SELENOP | Human | Rabbit | 1:500 | Santa Cruz Biotechnology(Santa Cruz, CA) |
| SELENOW | chicken | Rabbit | 1:1000 | Gift from Dr.Xu (Northeast agricultural university) |
| SELENON | chicken | Rabbit | 1:500 | Gift from Dr.Xu (Northeast agricultural university) |
| MSRB1 | Human | Rabbit | 1:1000 | Santa Cruz Biotechnology(Santa Cruz, CA) |
| p38 | Human | Rabbit | 1:1000 | Cell Signaling Technology (Beverly, MA) |
| p-p38 | Human | Rabbit | 1:1000 | Cell Signaling Technology (Beverly, MA) |
| JNK | Human | Rabbit | 1:1000 | Cell Signaling Technology (Beverly, MA) |
| p-JNK | Human | Rabbit | 1:1000 | Cell Signaling Technology (Beverly, MA) |
| ERK | Human | Rabbit | 1:1000 | Cell Signaling Technology (Beverly, MA) |
| p-ERK | Human | Rabbit | 1:1000 | Cell Signaling Technology (Beverly, MA) |
| RIPK1 | Human | Rabbit | 1:1000 | Cell Signaling Technology (Beverly, MA) |
| RIPK3 | Mouse | Rabbit | 1:1000 | Cell Signaling Technology (Beverly, MA) |
| MLKL | Human | Rabbit | 1:500 | Boster Biotechnology (Wuhan, China) |
| ACTB | Human | Rabbit | 1:3000 | Biosynthesis Biotechnolog y(Beijing, China) |

GPX1, glutathione peroxidase 1; GPX3, glutathione peroxidase 3; GPX4, glutathione peroxidase 4; SELENOP, selenoprotein P; SELENOW, selenoprotein W; SELENON, selenoprotein N; MSRB1, selenoprotein X; p38, p38 mitogen-activated protein kinase; p-p38, phospho-p38; JNK, c-Jun N-terminal kinase; p-JNK, phospho-JNK; ERK, extracellular signal-regulated kinase; p-ERK, phospho-ERK; RIPK1, receptor interacting serine threonine kinase 1; RIPK3, receptor interacting serine threonine kinase 3; MLKL, mixed lineage kinase domain-like; ACTB, β-acting.
